# Occupational Distribution of Campylobacteriosis and Salmonellosis Cases — Maryland, Ohio, and Virginia, 2014

**DOI:** 10.15585/mmwr.mm6632a4

**Published:** 2017-08-18

**Authors:** Chia-ping Su, Marie A. de Perio, Kathleen Fagan, Meghan L. Smith, Ellen Salehi, Seth Levine, Karen Gruszynski, Sara E. Luckhaupt

**Affiliations:** ^1^Epidemic Intelligence Service, CDC; ^2^Division of Surveillance, Hazard Evaluations, and Field Studies, National Institute for Occupational Safety and Health, CDC; ^3^Office of Occupational Medicine & Nursing, Occupational Safety and Health Administration, Washington, DC; ^4^CDC/Council of State and Territorial Epidemiologists, Applied Epidemiology Fellowship Program; ^5^Maryland Department of Health and Mental Hygiene; ^6^Ohio Department of Health; ^7^Virginia Department of Health.

*Campylobacter* and *Salmonella* are leading causes of bacterial gastroenteritis in the United States and are estimated to cause >1 million episodes of domestically acquired illness annually (*1*). *Campylobacter* and *Salmonella* are primarily transmitted through contaminated food, but animal-to-human and human-to-human transmission can also occur (*2**,**3**)*. Although occupationally acquired infections have been reported, occupational risk factors have rarely been studied. In 2015, the Occupational Safety and Health Administration (OSHA) identified 63 suspected or confirmed cases of *Campylobacter *infection over 3.5 years at a poultry-processing plant (Kathleen Fagan, OSHA, personal communication, December 2015); most involved new workers handling chickens in the “live hang” area where bacterial contamination is likely to be the highest. These findings were similar to those of a previous study of *Campylobacter* infections among workers at another poultry-processing plant (*4*). The investigation led to discussions among OSHA, state health departments, and CDC’s National Institute for Occupational Safety and Health (NIOSH); and a surveillance study was initiated to further explore the disease incidence in poultry-processing plant workers and identify any additional occupations at increased risk for common enteric infections. Deidentified reports of campylobacteriosis and salmonellosis among Maryland, Ohio, and Virginia residents aged ≥16 years were obtained and reviewed. Each employed patient was classified into one of 23 major occupational groups using the 2010 Standard Occupational Classification (SOC) system.* Risk ratios (RR) and 95% confidence intervals (CI) for associations between each occupational group and each disease were calculated to identify occupations potentially at increased risk, contrasting each group with all other occupations. In 2014, a total of 2,977 campylobacteriosis and 2,259 salmonellosis cases were reported. Among the 1,772 (60%) campylobacteriosis and 1,516 (67%) salmonellosis cases in patients for whom occupational information was available, 1,064 (60%) and 847 (56%), respectively, were employed. Persons in farming, fishing, and forestry as well as health care and technical occupations were at significantly increased risk for both campylobacteriosis and salmonellosis compared with all other occupations. Targeting education and prevention strategies could help reduce disease, and improving the systematic collection of occupational information in disease surveillance systems could provide a better understanding of the extent of occupationally acquired diseases.

For this analysis, deidentified reports of confirmed, probable, and suspected campylobacteriosis and salmonellosis^†^ cases reported during 2014 in residents aged ≥16 years were obtained from notifiable diseases surveillance systems in Maryland, Ohio, and Virginia. These states were invited to join in this study because occupation was recorded in a free text field in each case report in these states. In Ohio and Virginia, the reports also noted whether the patient was a health care worker, food handler, or daycare worker. Patients were assigned to one of three categories: employed, not employed (e.g., retired, student, homemaker, or unemployed at the time of disease reporting), or unknown. A standard two-digit 2010 SOC code was manually assigned to each case in an employed person. Where necessary, the NIOSH Industry and Occupation Computerized Coding System^§^ was used to assist in translating occupation text into standardized codes. Cases in persons in the military and those with occupations that could not be assigned a code because of insufficient information were excluded.

The 2014 American Community Survey (ACS)^¶^ was used to estimate the employed civilian population in the three included states combined. ACS, an ongoing survey, provides vital information about the U.S. population by state each year. RRs for each disease among each occupational group were calculated by comparing the risk for infection in each occupational group with risk among all other employed persons; 95% CIs were estimated based on a Poisson distribution using statistical software to conduct the analyses. 

In 2014, a total of 2,977 campylobacteriosis and 2,259 salmonellosis cases were reported in persons aged ≥16 years in Maryland, Ohio, and Virginia. Information about occupation was available for 1,772 (60%) campylobacteriosis cases and 1,516 (67%) salmonellosis cases. Among these, 1,064 (60%) campylobacteriosis patients and 845 (56%) salmonellosis patients were employed, and 708 (40%) and 669 (44%), respectively, were not employed (Figure).The 2014 ACS data for these three states combined indicated that 61% of persons aged ≥16 years were employed and 39% were not employed.

**FIGURE:**
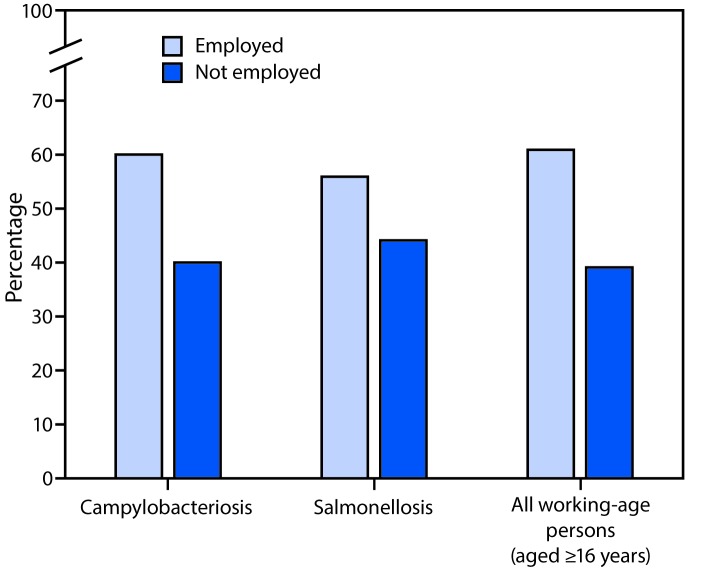
Percentage of campylobacteriosis and salmonellosis cases, and of all persons aged ≥16 years, by employment status — notifiable disease surveillance systems and American Community Survey, Maryland, Ohio, and Virginia, 2014

Among all cases in employed persons, nearly 72% of campylobacteriosis and 97% of salmonellosis cases were confirmed (Table 1). Compared with persons in other occupations, workers in farming, fishing, and forestry occupations and health care and technical occupations, were at significantly increased risk for campylobacteriosis (RR = 10.0 and 1.5, respectively) and salmonellosis (RR = 3.2 and 2.0) (Table 2). These two occupational groups accounted for 3.1% and 9.2% of campylobacteriosis cases and 1.0% and 11.5% of salmonellosis cases, respectively (Table 2). Workers in the broad category of production occupations were at increased risk for campylobacteriosis (RR = 1.4). A higher risk for salmonellosis was observed in workers in the food preparation and serving–related occupations (RR = 1.6) and personal care and service occupations (RR = 1.5). Among 41 campylobacteriosis cases among poultry-processing plant workers, cases occurred in three occupation categories: 38 in production, one in management, and two in building cleaning. 

**TABLE 1:** Characteristics of employed persons with campylobacteriosis (N = 1,064) and salmonellosis (N = 847) — notifiable diseases surveillance systems, Maryland, Ohio, and Virginia, 2014

Characteristic	No. (%)	
	Campylobacteriosis	Salmonellosis
**Case classification**		
Confirmed	761 (71.5)	822 (97.0)
Suspected	286 (26.9)	7 (0.8)
Probable	17 (1.6)	18 (2.1)
**Sex**		
Male	592 (55.6)	362 (42.7)
Female	472 (44.4)	485 (57.3)
**Age group (yrs)**		
16–24	119 (11.2)	133 (15.7)
25–44	409 (38.4)	369 (43.6)
45–64	450 (42.3)	284 (33.5)
≥65	83 (7.8)	59 (7.0)
Unknown	3 (0.3)	2 (0.2)
**Race/Ethnicity**		
White, non-Hispanic	766 (72.0)	608 (71.8)
Black, non-Hispanic	78 (7.3)	90 (10.6)
Hispanic/Latino	35 (3.3)	25 (3.0)
Asian, non-Hispanic	23 (2.2)	12 (1.4)
Other, non-Hispanic	4(0.4)	7 (0.8)
Unknown	158 (14.8)	105 (12.4)

**TABLE 2:** Distribution of all employed persons, campylobacteriosis* and salmonellosis cases,^†^ and calculation of relative risk for disease based upon occupational distributions,^§^ by occupational category — notifiable disease surveillance systems and American Community Survey, Maryland, Ohio, and Virginia, 2014

Occupation category	All employed	Campylobacteriosis		Salmonellosis	
	%	No. (%)	RR^¶^ (95% CI)	No. (%)	RR^¶^ (95% CI)
Management**	10.8	83 (8.5)	0.8 (0.6–1.0)	73 (9.2)	0.8 (0.7–1.1)
Business and financial operations	5.4	51 (5.2)	1.0 (0.7–1.3)	34 (4.3)	0.8 (0.6–1.1)
Computer and mathematical	3.9	42 (4.3)	1.1 (0.8–1.5)	26 (3.3)	0.8 (0.6–1.2)
Architecture and engineering	1.9	19 (2.0)	1.0 (0.6–1.6)	11 (1.4)	0.7 (0.4–1.3)
Life, physical, and social science	1.1	12 (1.2)	1.1 (0.6–1.9)	7 (0.9)	0.8 (0.4–1.6)
Community and social services	1.7	22 (2.3)	1.3 (0.9–2.0)	13 (1.6)	1.0 (0.6–1.7)
Legal	1.3	13 (1.3)	1.0 (0.6–1.8)	10 (1.3)	1.0 (0.5–1.8)
Education, training, and library	6.1	52 (5.3)	0.9 (0.7–1.2)	39 (4.9)	0.8 (0.6–1.1)
Arts, design, entertainment, sports, and media	1.9	20 (2.1)	1.1 (0.7–1.7)	12 (1.5)	0.8 (0.5–1.4)
Healthcare practitioners and technical**^††^**	6.2	90 (9.2)	1.5 (1.2–1.9)	92 (11.5)	2.0 (1.6–2.5)
Healthcare support	2.3	20 (2.1)	0.9 (0.6–1.4)	26 (3.3)	1.4 (1.0–2.1)
Protective service	2.4	24 (2.5)	1.0 (0.7–1.5)	23 (2.9)	1.2 (0.8–1.8)
Food preparation and serving related**^††^**	5.6	58 (6.0)	1.1 (0.8–1.4)	68 (8.5)	1.6 (1.2–2.0)
Building and grounds cleaning and maintenance**^§^**^§^	3.6	37 (3.8)	1.1 (0.8–1.5)	30 (3.8)	1.1 (0.7–1.5)
Personal care and service**^††^**	3.2	36 (3.7)	1.1 (0.8–1.6)	39 (4.9)	1.5 (1.1–2.1)
Sales and related	9.8	93 (9.6)	1.0 (0.8–1.2)	63 (7.9)	0.8 (0.6–1.0)
Office and administrative support	12.8	103 (10.6)	0.8 (0.7–1.0)	96 (12.0)	0.9 (0.8–1.2)
Farming, fishing, and forestry**^††^**	0.3	30 (3.1)	10.0 (7.0–14.4)	8 (1.0)	3.2 (1.6–6.4)
Construction and extraction	4.6	28 (2.9)	0.6 (0.4–0.9)	30 (3.8)	0.8 (0.6–1.2)
Installation, maintenance, and repair	3.1	29 (3.0)	1.0 (0.7–1.4)	17 (2.1)	0.7 (0.4–1.1)
Production**^††,^**^¶¶^	5.8	79 (8.1)	1.4 (1.1–1.8)	42 (5.3)	0.9 (0.7–1.2)
Transportation and material moving	6.2	32 (3.3)	0.5 (0.4–0.7)	37 (4.6)	0.7 (0.5–1.0)
**Total**	**100.0**	**973 (100.0)**	**—**	**796 (100.0)**	**—**

**Abbreviations:** CI = confidence interval; RR = relative risk.

* Excludes 75 employed persons with unclassified occupation and 16 in active military service.

^†^ Excludes 40 employed persons with unclassified occupation and 11 in active military service.

^§^ Calculated using data from the American Community Survey.

^¶^ Risk for infection in each occupation divided by that among all other employed persons.

** Includes 13 self-employed farmers or farm owners with campylobacteriosis and six with salmonellosis; one poultry-processing plant manager with campylobacteriosis.

**^††^** Occupation categories for which the 95% CI for the RR for one or both diseases does not include 1.0.

**^§^**^§^ Includes two sanitation workers in poultry-processing plants with campylobacteriosis and one with salmonellosis.

^¶¶^ Includes 38 poultry-processing plant workers with campylobacteriosis and three with salmonellosis.

## Discussion

This report describes the occupational distribution of campylobacteriosis and salmonellosis cases in three states during 2014. Persons in farming, fishing, and forestry occupations and health care and technical occupations were at increased risk for both campylobacteriosis and salmonellosis. The food preparation and serving–related occupations and personal care and service occupations were also at higher risk for salmonellosis. Although *Campylobacter* and *Salmonella* infections are typically considered foodborne, both have other potential sources such as ill patients, animals, and the environment. The incidence of foodborne illnesses, including those attributable to *Campylobacter* and *Salmonella*, has changed little despite recent improvements in food safety (*1*). Targeting of education and prevention strategies (e.g., disease awareness and proper hand hygiene techniques at work) toward specific groups at high risk and their employers could help reduce the incidence.

A recent systematic literature review found that certain occupational groups, including health care workers and workers with animal contact, are at increased risk for exposure to work-related infectious diseases (*3*). Therefore, occupational information could be important in identifying groups at increased risk for enteric infections. In addition, occupational information could be used to examine the contribution of work-related environmental hazards, including infectious pathogens, to explain different risks for health outcomes in the United States (*5*). Nevertheless, the occupational information in current infectious disease surveillance systems is inadequate and has rarely been analyzed systematically to describe patterns of disease by occupation.

The finding that agriculture workers are at higher risk for infection is not surprising because of the opportunities for exposure and potential for disease transmission in the workplace. An estimated 17% of campylobacteriosis and 11% of salmonellosis cases are attributable to animal contact (*6*)*,* and contact with farm animals previously has been identified as a risk factor for sporadic *Campylobacter* infection in the United States (*7*). The current analysis also showed campylobacteriosis cases among workers with different duties in multiple poultry-processing facilities, supporting the previous finding that poultry workers are at elevated risk for *Campylobacter* exposure because of heavy workplace contamination (*8*).

Health care workers, personal care and service workers, and food preparation workers were also found to be at increased risk for infection. *Campylobacter* and *Salmonella* can also be transmitted from person to person by the fecal-oral route. Therefore, health care workers might be exposed to these pathogens through contact with patients, which indicates a potential occupational risk. Occupational transmission of *Salmonella* to health care workers has been previously identified (*3*). Occupationally acquired *Campylobacter* infections among health care workers are also possible, but have not been described. The personal care and service occupations category includes certain occupations involving close contact with patients in long-term care facilities and children in child care settings. Persons who care for nontoilet-trained children are known to be at risk for contact with enteric pathogens (*9*). Additionally, workers in food preparation and serving-related occupations might be at increased risk for salmonellosis from handling contaminated meat or foods and are known to be sources of transmission in outbreaks (*10*). Because of the risk for spread of the disease to customers or clients, all cases of campylobacteriosis and salmonellosis among workers should be reported and reviewed to identify the source and prevent ongoing transmission.

The findings in this report are subject to at least three limitations. First, employment in an occupation at high risk for infection does not prove causation; other possible exposure sources were not evaluated. Risk factors among specific workers must be studied to better characterize the risk for occupationally acquired diseases. Second, occupational information was missing for multiple cases and data might not be missing at random. Cases in the three job categories with specific fields on case report forms (i.e., health care worker, food handler, or daycare worker) might have been more likely to be recorded. Finally, despite a combination of manual and computer-assisted occupation coding processes, misclassification might have occurred because of incomplete descriptions and the absence of a field for industry on the case reports. In general, the term “industry” refers to the type of business for which a person works (e.g., poultry-processing plant), and the term “occupation” refers to a worker’s specific job (e.g., plant manager). The collection of both industry and occupation information can help public health workers identify potential risk factors in need of further assessment.

Campylobacteriosis or salmonellosis should be considered when workers in occupations at increased risk for infection have symptoms compatible with these diseases. Discovering underlying mechanisms of transmission and assessing hazards in the workplace could help employers plan disease prevention measures, such as providing personal protective equipment and hand hygiene education. To improve data collection in surveillance systems, occupational questions should be standardized, information on both industry and occupation should be collected, and data should be analyzed with standard coding schemes to monitor disease trends in specific industries or occupations and protect workers’ health.

**Summary**

**What is already known about this topic?**

*Campylobacter* and *Salmonella* are leading causes of bacterial gastroenteritis in the United States with >1 million cases reported annually. These pathogens are primarily transmitted through consumption of contaminated food, but animal-to-human and human-to-human transmission also occur. Occupational transmission has been reported, but there is limited information regarding patterns of disease by occupation.

**What is added by this report?**

In 2014, 2,977 campylobacteriosis and 2,259 salmonellosis cases were reported in Maryland, Ohio, and Virginia; 1,064 (60%) and 847 (56%) patients, respectively, were employed. Persons in farming, fishing, and forestry occupations and health care and technical occupations were at increased risk for both campylobacteriosis and salmonellosis. Persons in food preparation and serving–related occupations and personal care and service occupations were also at higher risk for salmonellosis.

**What are the implications for public health practice?**

Increased risk for enteric infection among workers in agriculture, health care, food, and personal care occupations might be related to workplace exposures to pathogens. Campylobacteriosis or salmonellosis should be considered when workers have symptoms compatible with these diseases. Targeting education and prevention strategies, including disease awareness and proper hygiene techniques at work, to groups at higher risk and their employers could help reduce disease.

## Footnotes

*
https://www.bls.gov/soc/ .

† According to the 2012 Council of State and Territorial Epidemiologists’ case definitions. https://wwwn.cdc.gov/nndss/case-definitions.html.

§
https://wwwn.cdc.gov/niosh-nioccs/ .

¶
https://www.census.gov/programs-surveys/acs/ .
